# Antimicrobial Resistance in Equine Reproduction

**DOI:** 10.3390/ani11113035

**Published:** 2021-10-22

**Authors:** Pongpreecha Malaluang, Elin Wilén, Johanna Lindahl, Ingrid Hansson, Jane M. Morrell

**Affiliations:** 1 Clinical Sciences, Swedish University of Agricultural Sciences (SLU), Box 7054, SE-75007 Uppsala, Sweden; pongpreecha.malaluang@slu.se (P.M.); elin.wilen@outlook.com (E.W.); johanna.lindahl@imbim.uu.se (J.L.); 2 Department of Biosciences, International Livestock Research Institute, P.O. Box 30709, Nairobi 00100, Kenya; 3 Department of Medical Biochemistry and Microbiology, Uppsala University, Box 582, 75123 Uppsala, Sweden; 4 Biomedical Science and Veterinary Public Health, Swedish University of Agricultural Sciences (SLU), Box 7036, SE-75007 Uppsala, Sweden; ingrid.hansson@slu.se

**Keywords:** antibiotics, resistance mechanisms, sperm quality, uterine health, semen extenders, prudent use of antimicrobials

## Abstract

**Simple Summary:**

Bacteria can develop resistance to antibiotics, resulting in the appearance of infections that are difficult or impossible to treat. This ability enables bacteria to survive in hostile environments and can result from exposure to even small amounts of antibiotic substances. Bacteria are present in the reproductive tract of the horse; they can develop resistance to antibiotics, because the animal has been treated for an infection, or due to insemination with a semen dose that contains antibiotics. Bacteria colonize the membrane lining the male reproductive tract and are transferred to the semen during collection. They can cause sperm quality to deteriorate during storage or may cause an infection in the mare. Therefore, antibiotics are added to the semen dose, according to legislation. However, these antibiotics may contribute to the development of resistance. Current recommendations are that antibiotics should only be used to treat bacterial infections and where the sensitivity of the bacterium to the antibiotic has first been established. Therefore, adding antibiotics to semen extenders does not fit these recommendations. In this review, we examine the effects of bacteria in semen and in the inseminated mare, and possible alternatives to their use.

**Abstract:**

Bacteria develop resistance to antibiotics following low-level “background” exposure to antimicrobial agents as well as from exposure at therapeutic levels during treatment for bacterial infections. In this review, we look specifically at antimicrobial resistance (AMR) in the equine reproductive tract and its possible origin, focusing particularly on antibiotics in semen extenders used in preparing semen doses for artificial insemination. Our review of the literature indicated that AMR in the equine uterus and vagina were reported worldwide in the last 20 years, in locations as diverse as Europe, India, and the United States. Bacteria colonizing the mucosa of the reproductive tract are transferred to semen during collection; further contamination of the semen may occur during processing, despite strict attention to hygiene at critical control points. These bacteria compete with spermatozoa for nutrients in the semen extender, producing metabolic byproducts and toxins that have a detrimental effect on sperm quality. Potential pathogens such as *Klebsiella pneumoniae*, *Escherichia coli*, and *Pseudomonas aeruginosa* may occasionally cause fertility issues in inseminated mares. Antibiotics are added during semen processing, according to legislation, to impede the growth of these microorganisms but may have a detrimental effect on sperm quality, depending on the antimicrobial agent and concentration used. However, this addition of antibiotics is counter to current recommendations on the prudent use of antibiotics, which recommend that antibiotics should be used only for therapeutic purposes and after establishing bacterial sensitivity. There is some evidence of resistance among bacteria found in semen samples. Potential alternatives to the addition of antibiotics are considered, especially physical removal separation of spermatozoa from bacteria. Suggestions for further research with colloid centrifugation are provided.

## 1. Introduction

The World Health Organisation (WHO) described antibiotic resistance as being one of the greatest challenges facing humankind in the modern world [1]. There are already many instances where bacterial infections, such as tuberculosis, pneumonia, food-borne diseases, or gonorrhoea, do not respond to treatment with currently available antibiotics [2]. No new classes of antimicrobial agents have been developed in the last few decades, and there is no incentive for pharmaceutical companies to invest to produce new ones [3]. Even low-level usage of antibiotics, such as in topical applications, can contribute to the appearance of antimicrobial resistance (AMR). Therapeutic treatment of both animals and humans contributes to background resistance, i.e., maintaining resistance within bacteria in the environment. Thus, in an effort to curb the development of AMR, antibiotics should be used only for therapeutic purposes and preferably after sensitivity testing of the bacteria present [4].

Although AMR in horses has been reported previously, the subject of AMR in the reproductive tract of horses has not been described in detail. The widespread use of antibiotics in semen extenders used to prepare semen doses for artificial insemination (AI) could represent an important cause of AMR in brood mares, and therefore deserves particular attention. This application represents a hidden use of antibiotics that may not be completely justified. Therefore, this review presents an overview of AMR in the reproductive tract of the mare. First, we will consider how bacteria develop resistance, since this is crucial to any attempt to hinder AMR, followed by a description of how bacteria colonize the reproductive tract of the mare. In the next section, we describe how these bacteria are exposed to antibiotics and the effects of the antibiotics on sperm quality and on the mare. Finally, suggestions for alternatives to antibiotics to delay the development of AMR are presented. The search terms are shown in Supplementary file.

## 2. Development of Antimicrobial Resistance

Bacteria may have natural resistance to different types of antibiotics, or they acquire it. Resistance can be due to genetic mutations or due to selection pressure in nature and can arise in both pathogenic and non-pathogenic bacteria. A variety of resistance genes occur in environmental bacteria [5]. Exposure to antibiotics facilitates the development of AMR, since resistant bacteria have an advantage over other strains in the same environment. This selection also explains why topical use of antibiotics contributes to AMR. Opinions differ on how much exposure to antibiotics is needed for resistance to develop. A study of *Clostridium perfringens* concluded that even small amounts of antibiotics could result in resistance [6]. Another study, however, suggested that a higher concentration of antibiotics is required to induce AMR than is usually seen in a non-clinical environment, although it can arise due to pollution events [7], such as incorrect disposal of antibiotic-containing substances into the environment. The duration of exposure needed to induce AMR is likely to be different for different antibiotics, but is poorly defined [7].

**Figure 1:**
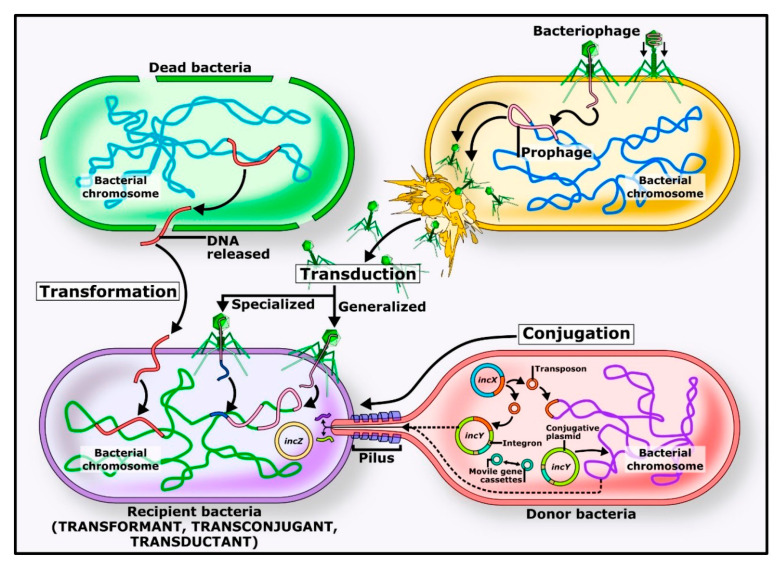
Mechanisms of horizontal gene transfer in bacteria [10]. Note: The main routes of transmission of genetic material between bacteria are transformation, transduction and conjugation. Genes can pass between related and non-related species by these routes. These mechanisms of transmission allow bacteria to evolve to survive.

Genes for acquired resistance can be transferred between bacteria [8], allowing the bacteria to thrive in an otherwise toxic environment. The main horizontal means of transmission in nature are by conjugation, transduction, and transformation [9], as shown in Figure 1. During conjugation, transfer of the genetic sequence in a plasmid occurs between in-contact bacteria through a tube-like structure known as a pilus. Some, mainly Gram-positive, bacteria can conjugate via transponders [9]. In transduction, bacteriophages transfer genetic material between bacteria, although this is a relatively ineffective mode of transmission, infecting only closely related species of bacteria [9]. With transformation, exogenous DNA is absorbed into the bacterial cell through a specific membrane channel and incorporated into the bacterial DNA [10]. Even dead bacteria can pass on resistance genes to other bacteria by this method [10].

Once acquired, resistance genes are transmitted vertically during cell division. Enterococci are able to transfer AMR to less resistant strains [11], and *Aeromonas* spp. can pass resistance genes to unrelated bacteria [12]. Transfer of plasmids enables other DNA, e.g., obtained via transformation, to be passed on to subsequent generations. Therefore, use of one antimicrobial agent can lead to the transfer of resistance against other antimicrobials within the population [13].

Acquisition of AMR has a fitness cost [14], since bacteria with resistance genes start to produce novel genetic elements at a higher metabolic cost than the non-resistant cell. In the absence of selection pressure, the genes for resistance will be lost due to competition from bacteria without the gene [9]. Thus, resistance patterns within any given bacterial population are evolving constantly, corresponding to selection in response to changes in the environment.

An isolate is considered to be resistant if it has a higher minimum inhibitory concentration (MIC) than the corresponding wild-type strain [15]. Different categories of resistance occur: bacteria with acquired resistance to antimicrobial substances from three or more antibiotic classes are known as multi-drug resistant; if susceptible to one or two antimicrobial substances, they are defined as extensively drug-resistant; bacteria that are non-susceptible to all agents in all antimicrobial classes are pan-drug-resistant [16]. Examples of the latter are strains of *Klebsiella (K) pneumoniae* and *Acinetobacter* spp. [16], both of which are reported to be common constituents in the vaginal flora of the mare [17].

Risk factors for the development of AMR in the flora of the reproductive tract include repeated use of antibiotics, e.g., against infections, but also their non-therapeutic use in semen extenders [18,19]. Bacteria are found on the mucosa of the distal reproductive tract of healthy stallions and are transferred to the ejaculate during semen collection [20] or may contaminate the ejaculate from the environment during processing. Adding a semen extender not only supplies nutrients and buffers to keep the spermatozoa alive but also provides an excellent medium for the growth of microorganisms. Therefore, antibiotics are added to semen extenders to prevent deterioration of sperm quality to be used are specified in various regulations, e.g., as stipulated by European Council Directive 92/65/EEC [21]. In addition, strict hygiene measures are taken to reduce bacterial contamination during semen collection and artificial insemination (AI) and stallions should not be used for both natural mating and semen collection for AI during the same time period [22]. Bacteria in sperm samples will be discussed in detail in Section 4.

## 3. Bacteria in the Equine Uterus

Although the healthy equine uterus was previously considered to be sterile, it is now known to possess its own flora [23]. These observations have arisen from the use of bacterial DNA sequencing (16S sequencing), enabling bacteria to be identified that would not appear on culture [24]. Some mares have poor conformation of the vulva or perineal region [25], predisposing them to additional bacterial contamination of the reproductive tract. Such poor conformation can be inherited or occur with age. Injury to the cervix and/or vagina may predispose to bacterial ingress, whereas poor uterine clearance predisposes to persistence of bacteria within the uterus. Bacteria in the caudal reproductive tract (vagina and cervix) may be carried into the uterus during mating or AI, or during gynecological examination despite strict hygienic measures [26]. The effect of bacteria in semen will be considered in detail in Section 5.

Occasionally, the bacteria introduced during mating or insemination are pathogenic. Thus, *Streptococcus* (*Str.) equi* ssp. *zooepidemicus*, *K pneumoniae*, *Escherichia* (*E.*) *coli* and *Pseudomonas (Ps.) aeruginosa* have been found in the urethra and prepuce of stallions after mating (51.1%, 28.9%, 17.8% and 2.2%, respectively) [27]. These bacteria were also isolated from mares bred by these stallions (90.9%, 1.3%, 1.3%, and 1.3%, respectively), indicating that the stallion was the source of some of the bacteria. There is a well-defined response in the reproductive tract of the mare to deal with bacteria (and other debris) deposited during mating [28]. However, this response may be overwhelmed if the bacterial load is very high or if specific bacteria are present, which are known pathogens. These bacteria are *Taylorella equigenitalis* (the causative agent of contagious equine metritis, CEM), *Klebsiella pneumoniae*, and *Ps aeruginosa* [29]. In addition, *Str. equisimilis, Str. zooepidemicus, E. coli, Bacillus* spp. [30,31], *Mycoplasma (M.) equigenitalium, M. subdolum*, and *Acholeplasma* spp. have been isolated from infertile mares or from mares diagnosed with endometritis or abortion [32,33], although these organisms may be facultative pathogens rather than obligate pathogens.

An inflammatory response occurs following mating, involving cytokines and complement [34]. Its function is to remove bacteria, seminal plasma, and excess spermatozoa from the uterus [25]. Fluid and inflammatory cells are normally voided within 24–48 h after mating. However, in some mares, there is retention of fluid and inflammatory cells for prolonged periods, which affects ciliary function and leads to the development of acute endometritis, with bacteria adhering to the endometrium. Vascular degeneration occurs and uterine drainage is disrupted, reducing venous return to capillary beds [34] and disturbing hormone delivery to the endometrium. The presence of lymphocytes and plasma cells for prolonged periods leads to chronic degeneration in the endometrium. It is not known whether bacteria are the primary initiators of these events or are secondary invaders of the affected tissue [35].

## 4. Antimicrobial Resistance in the Mare’s Reproductive Tract

**Figure 2:**
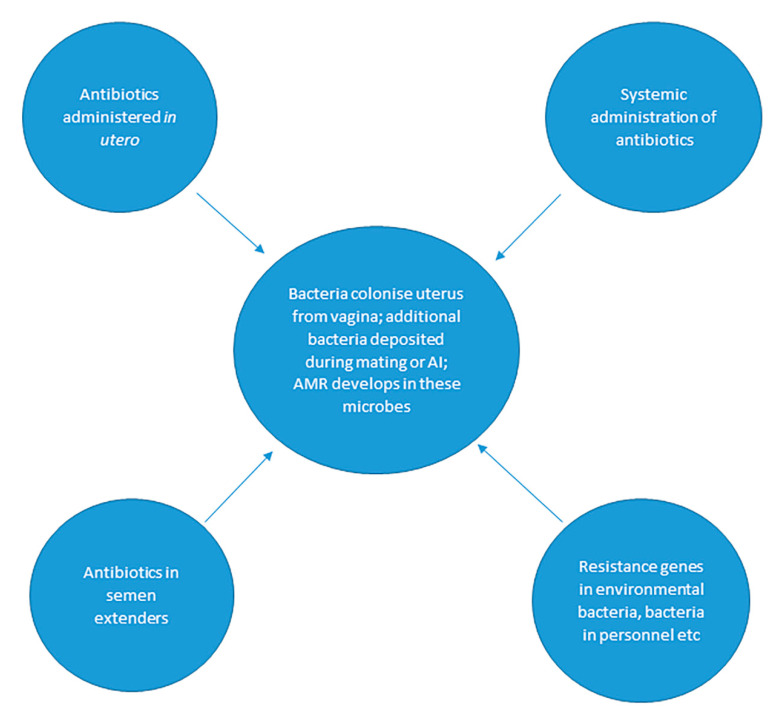
Sources of AMR in the microbial flora of the equine reproductive tract. Note that AMR can develop by any of the mechanisms described in Section 2.

The bacterial flora of the mare’s reproductive tract is exposed to antibiotics via several routes: after systemic administration, via local application and in semen extenders used in AI. The drivers for the development of AMR are summarized in Figure 2.

Some antibiotics are found in the uterus after systemic administration; for example, ceftiofur administered systemically passed from the bloodstream to the uterus [36]. The penetration of antibiotics into uterine tissue and secretions depends on the pharmacodynamics of the particular compound, the dose and route of administration, and duration of exposure. Concentrations of sulfadiazine-trimethoprim administered orally were reported to exceed MIC in the endometrium of healthy mares [37]. Enrofloxacilin given by the intravenous route was effective against uterine infections, i.e., achieved effective concentrations in uterine tissue [38], whereas it was not recommended for uterine lavage due to tissue irritation [39]. Regarding transfer from the uterus to the bloodstream, ciprofloxacin administered by the intrauterine route achieved MICs in uterine tissue in healthy mares but with minimal systemic absorption [40]. In contrast, neomycin administered by the intrauterine route was transferred to the blood stream, although the uptake depended on the stage of the oestrous cycle and whether or not infection was present [41]. It should be noted that most of the studies that have been performed on transfer of antibiotics to the uterus after systemic administration or between the uterus and the rest of the body after intrauterine administration have been carried out in healthy animals [35]; the pharmacodynamics may be different in the presence of pathology. Studies in cattle report contradictory findings regarding penetration of antibiotics into the uterus following systemic administration. Thus, ceftiofur administration to cows with postpartum metritis did not influence the number or type of bacteria present in the uterus but did reduce the number of days of elevated body temperature in these animals [42]. Oxytetracycline administered intramuscularly to cows with metritis resulted in penetration of the antibiotic into uterine secretions, but levels were below MIC values [43]. Therefore, apparently some antibiotics in the insemination dose can pass from the uterus to the bloodstream. Local application includes uterine administration, e.g., for endometritis, but also occurs via inseminated semen, in which antibiotics are added to the semen extender. Either local or systemic administration of antibiotics could therefore lead to development of AMR in the bacterial flora of the reproductive tract via the mechanisms already described, even through low-level exposure.

Several articles from various countries report AMR in bacteria from the equine reproductive tract; the bacteria were isolated from uterine lavage or swabs from the uterus, vagina, or clitoris. The reports indicate that AMR is widespread in the bacteria isolated from this material, regardless of the reproductive status of mares, although mares with reproductive problems, such as failure to conceive, were more commonly sampled than those with normal fertility. The findings of the various reports are summarized in Table 1. Longitudinal studies in some countries indicate that the AMR pattern has changed over time, e.g., in the United States [26] and Italy [44]. However, it is not clear whether the increase in reporting is due to an actual increase in occurrence or merely a reflection of wider testing.

## 5. Bacteria in Semen

Bacteria from the skin and from the animal´s environment colonize the mucosa of the distal reproductive tract and are transferred to the semen during ejaculation [54]. The majority of these bacteria are non-pathogenic but may be facultative pathogens or pathogens, as previously described (Section 3). Therefore, semen collection should be conducted with strict attention to hygiene. However, washing the penis prior to semen collection to remove superficial dirt and debris is controversial, since it may remove the normal skin flora, predisposing it to overgrowth by other bacteria [55]. In any case, this procedure is unlikely to remove bacteria from the mucosa. However, some authors have reported fewer bacteria in semen following penile washing [56]. Differences between studies regarding bacterial contamination may reflect the environment in which the stallions are kept [57], in addition to factors such as the number of intromissions into the artificial vagina required before ejaculation is completed.

As explained previously, the presence of some bacteria in semen is not a problem for the mare per se, unless large numbers or specific types of bacteria are present. However, AI is increasingly performed with cooled semen transported to other premises [58], allowing additional time for microbes to grow as the semen is being processed and cooled. Therefore, the bacterial load may be considerably greater by the time of AI than immediately after semen collection.

### 5.1. Effects of Bacteria on Sperm Quality

Bacteria compete with spermatozoa for nutrients in the extender, producing metabolic byproducts and toxins that have a detrimental effect on sperm quality. Bennett [59] reported a negative association between bacteria in stallion semen and both sperm quality and fertility. The bacterial load of *Klebsiella* spp. and *Pseudomonas* spp. in semen was negatively correlated with sperm membrane integrity [60], and *Klebsiella* spp. were correlated with the proportion of dead spermatozoa. Lateral sperm head displacement post-thaw was negatively correlated with the presence of non-β-haemolytic *streptococci* [60]. Other researchers reported that the microbial flora present in stallion semen was not linked to sperm quality [57].

In other species, bacteria belonging to the family *Enterobacteriaceae* in ram semen were associated with reduced sperm quality during storage at 15 °C [61]. The presence of bacteria was associated with reduced motility in human semen samples; another microorganism, the yeast *Candida albicans*, was found in azoospermic semen [62].

### 5.2. Effects of Antibiotics in Semen Extenders on Bacteria

Various combinations of antibiotics (potassium penicillin G-amikacin disulfate, ticarcillin disodium-potassium clavulanate, piperacillin sodium/tazobactam sodium, or meropenem) were moderately effective against low doses of *K. pneumoniae* or *Ps. aeruginosa* inoculated into semen. However, these antimicrobials did not inhibit growth when higher numbers of the bacteria were inoculated [63]. The presence of biofilms, such as in some cases of chronic endometritis, may render antibiotics ineffective.

### 5.3. Effects of Antibiotics in Semen Extenders on Sperm Quality

An extensive range of antimicrobial substances is used in semen extenders for various species [64]. These latter authors summarized the antibiotics used for stallion sperm samples, including amikacin, cefquinome, ceftiofur, clavulanic acid, gentamicin, meropenem, penicillin, and ticarcillin. However, antibiotics can have a negative effect on sperm quality, as summarized in Table 2.

## 6. Effects of Antibiotics in Semen Extenders on the Mare

The uterine microbiome is exposed to antibiotics each time the mare is inseminated. The liquid portion of the inseminate is expelled by backflow in the hours following insemination, exposing bacteria in the vagina and the environment to the antibiotics in the semen extenders. A study was conducted in Sweden to determine the effect of this exposure on the vaginal flora. Some changes in resistance patterns of vaginal bacteria were observed following exposure to antibiotics in a semen extender. Approximately half of the 1036 isolates identified were *E. coli*, and 13.3% were *Str. dysgalactiae*. There was an increase in resistance of *E. coli* to trimethoprim and chloramphenicol after AI, although there was no change in resistance to tigecycline or ampicillin. The resistance patterns of *Str. dysgalactiae* and *Staphylococcus (St.) simulans* isolates did not change after AI. *Str. dysgalactiae* isolates were resistant to erythromycin, nitrofurantoin, and tetracycline, whereas some *St. simulans* isolates were resistant to penicillin, oxacillin, and fusidic acid. None of the *Enterococcus faecalis* isolates were resistant to any of the antibiotics [69].

Apart from the effect on the vaginal flora of the mare, the personnel handling the semen doses may be exposed to antibiotics, although the risk is likely to be small if normal hygienic measures are taken. However, it is important that the remains of semen extenders or unused insemination doses be disposed of properly, not by pouring down the drain [46], to avoid further contamination of the environment and exposure of environmental bacteria to antimicrobials. Proper disposal involves boiling to destroy the antibiotic activity or placing in waste for burning [70].

## 7. Alternatives to Addition of Conventional Antibiotics to Semen Extenders

### 7.1. Antimicrobial Peptides

Antimicrobial peptides are produced by the immune system of some mammals as a defense against bacteria, as reviewed by Vickram et al. [71]. They are active against a range of microorganisms, depending on structural differences [72]. A unifying feature is that they are amphipathic and have a cationic charge; these features could help to explain their selective action on negatively charged lipids in bacterial membranes [73]. Although their use has not been reported in semen extenders for stallion semen, a cationic peptide derived from semenogelin was proposed as an antimicrobial agent for addition to extenders for human semen [74]. Another peptide, GL13K, was reported to be active against a biofilm containing *Ps. aeruginosa* [75]. A cyclic hexapeptide was considered to be a potential candidate as an antimicrobial agent for boar semen, as it apparently did not affect pregnancy rates in AI when used in combination with a low dose of gentamicin, in contrast to other peptides that negatively affected sperm membrane integrity [76]. More recently, magainin derivatives and cyclic hexapeptides were investigated for inclusion in boar semen extenders [77]. However, opinion is divided as to whether these antimicrobial peptides could provide activity against all microbes without provoking resistance [71].

### 7.2. Nanoparticles

The use of iron oxide (Fe_3_O_4_) nanoparticles during boar semen processing produced a slight antibiotic effect with no adverse effects on sperm characteristics [78]. However, the effect on stallion spermatozoa has not been reported.

### 7.3. Physical Removal of Bacteria

Microfiltration of boar seminal plasma through a syringe prefilter was reported to reduce bacterial load when sperm doses for artificial insemination were reconstructed with this seminal plasma [79]. However, such a method might not be practical for preparing large volumes of semen, such as whole stallion ejaculates, since it involves both centrifugation and filtering. As yet, there have not been any documented reports of its use for stallion semen.

Colloid centrifugation was used to separate spermatozoa from bacteria in an ejaculate. This technique is a relatively simple procedure and prolongs the survival of stallion spermatozoa without adding antibiotics in the semen extender [67,80]; it is practical for use on the stud farm, only requiring access to a centrifuge with a swing-out rotor. In single layer centrifugation (SLC), a colloid formulation with high density is poured into a centrifugation tube, and extended semen is carefully pipetted on top. The preparation is then centrifuged at 300× *g* for 20 min before removing the supernatant and most of the colloid. The sperm pellet is harvested using a sterile pipette [81]. It was shown that semen quality is improved in various species after SLC, including stallions, and the number of bacteria in the sample is considerably reduced. The SLC was effective in removing 81% to > 90% of bacteria from stallion semen, depending on bacterial load and species [80]. Similar results were found in a study by Al-Kass et al. [67], although interestingly, they found that 25% of the bacteria remained in the samples after SLC if antibiotics were present in the extender, whereas only 18% of the bacteria remained after SLC where no antibiotics were included. Further studies are underway using a low-density colloid to separate boar spermatozoa from seminal plasma with its bacterial load, without selecting for robust spermatozoa [82]; this method has not been tested with stallion semen yet.

## 8. Conclusions

Bacteria are present in the uterus of the mare; AMR in the flora of the mare´s reproductive tract is reported in many countries and involves resistance against several antimicrobial agents. It is not known whether AMR arises primarily from local antibiotic treatment or whether systemic administration also plays a significant role. Since certain antibiotics can appear in the uterus after systemic administration, it is likely that this route of administration does play a part in the development of AMR in the equine reproductive tract. However, a major route of exposure of the bacteria in this location is via antibiotics in semen extenders used in insemination doses. Thus, it is clear that the recommendations concerning prudent use of antimicrobials are also appropriate for any intra-uterine application of antibiotics, including AI. Since AMR can be spread by low-level usage of antibiotics, as well as by therapeutic administration, it would be advisable to avoid use of antibiotics whenever possible. The addition of antibiotics to semen extenders is a situation where alternative (physical) methods are available to remove bacterial contamination, thus obviating the need for antibiotics in this context. Colloid centrifugation is a practical method for reducing the bacterial load in semen samples and can be carried out effectively using equipment that is already present on many studs. The addition of antibiotics to semen extenders may not be the only way to impede bacterial growth, but further studies, including large-scale AI trials, are needed to ensure that fertility is not compromised by any remaining bacteria in insemination doses. Antimicrobial peptides or iron oxide nanoparticles could also offer useful alternatives to the addition of antibiotics, but testing of these compounds with stallion semen is necessary before their use in this species can be recommended.

## Figures and Tables

**Table 1:** Summary of antimicrobial resistance patterns in the equine reproductive tracts in various countries.

Country and Source	Material Used and Susceptibility/Resistance Results
France [45]	*Taylorella equigenitalis* isolates from cervical swabs of mares with acute endometritis or cervicitis; the isolates were resistant to streptomycin, clindamycin, lincomycin, and metronidazole.
Sweden [19]	135 bacterial isolates from uterine swabs from mares with fertility problems; ß-haemolytic *Streptococcus* was resistant to gentamicin, neomycin, oxytetracycline, and trimethoprim-sulfamethoxazole out of the 11 tested antibiotics. *E. coli* was resistant to 9 of 10 tested antibiotics, including ampicillin, cephalothin, chloramphenicol, gentamicin, neomycin, nitrofurantoin, oxytetracycline, streptomycin, and trimethoprim-sulfamethoxazole.
India [46]	Bacteria from uterine flushes from three repeat breeder mares included *Escherichia coli*, *Klebsiella* spp. and *Micrococcus* spp. susceptible to amoxicillin, chloramphenicol, doxycycline and gentamicin and resistant to cloxacillin, metronidazole, penicillin, and sulphadiazine.
Italy [47]	Isolates from uterus of mares with fertility problems: *Str. group C* were only susceptible to amoxicillin/clavulanic acid at 82.7%. *E. coli* showed high susceptibility to a significant number of drugs.
India [17]	Bacteria in vaginal swabs of infertile and healthy mares showed resistance. All isolates belonging to *Streptococcus* spp. were highly resistant to amoxiclav, ampicillin, carbenicillin, cefotaxime, cephalexin, enrofloxacin, clindamycin, cloxacillin, co-trimoxazole, co-trimazine, erythromycin, gentamicin, oxacillin, and tetracycline. *Enterococcus* spp. and *E. coli* isolates from infertile mares were resistant to ß-lactam antibiotics and imipenem. *Enterococcus* spp. were highly resistant to ampicillin, carbenicillin, cefdinir, cefotaxime, cephalexin, chloramphenicol, enrofloxacin, clindamycin, cloxacillin, co-trim-oxazole, co-trimazine, erythromycin, gentamicin, norfloxacin, oxacillin, and vancomycin.
US [26]	Uterine swab collected at pre-breeding examination or infertility investigation. *E. coli* was highly resistant to ampicillin and trimethoprim-sulfonamide, *S. equi* subsp. *zooepidemicus* was highly resistant to oxytetracycline and bacteria belonging to *Enterobacteriaceae* were highly resistant to ampicillin, cefazolin, penicillin, and polymyxin B.
Germany [48]	Isolates from the uterus of mares with fertility problems showed that ß-hemolytic *streptococci* were resistant to colistin, whereas all *E. coli* strains were resistant to penicillin and erythromycin.
Slovakia [49]	Bacterial pathogens in equine cervical swabs in English thoroughbred mares taken during the foal heat cycle. β-haemolytic *streptococci* and *K* spp. showed high resistance to penicillin. *E. coli, Pseudomonas* spp. were highly resistant to penicillin and sulfisoxazole, and *Proteus* spp. were highly resistance to penicillin, tetracycline and sulfisoxazole.
Turkey [50]	Endometrial swabs taken from mares with pneumovagina and normal mares. *E. coli* was resistant to penicillin. *S. equi* subsp. *zooepidemicus* was highly resistant to tetracycline and colistin. *Staphylococcus intermedius* was resistant to penicillin, tetracycline, erythromycin, gentamicin, and colistin. *Str. equinus* was highly resistance to enrofloxacin, gentamicin, and colistin. *Ent. faecium* was resistant to ceftiofur and enrofloxacin. *Gardnerella vaginalis* was highly resistant to gentamicin, and sulfamethoxazole/trimethoprim.
Italy [51]	*Ent. casseliflavus* isolates from a mare with endometritis were resistant or intermediate to 18 of the 23 tested antibiotics, including amikacin, kanamycin, neomycin, streptomycin, imipenem, meropenem, ceftiofur, ceftriaxone, ciprofloxacin, enrofloxacin, norfloxacin, clindamycin, erythromycin, amoxicillin-clavulanic acid, ampicillin, colistin sulfate, rifampicin, and trimethoprim-sulfamethoxazole.
US [52]	Gram-positive bacteria from mares with postpartum metritis were highly resistant to ampicillin, clarithromycin, clindamycin, erythromycin, oxacillin, penicillin, rifampin, ticarcillin, and trimethoprim/sulfonamides. *Str. zooepidemicus* was highly resistant to amikacin, enrofloxacin, and orbifloxacin.
Italy [44]	Isolates from uterine swabs of mares suffering from endometritis. *E. coli* was highly resistant to ampicillin, cefquinome, cefazolin, ceftiofur, penicillin, rifampin, and thiamphenicol. *Str. zooepidemicus* was highly resistant to amikacin, cefazolin, ceftiofur, enrofloxacin, gentamicin, and marbofloxacin.
India [53]	Isolates from cervical swabs of mares presented at the clinic. *E. coli* was sensitive to ofloxacin, azithromycin, gentamicin and amikacin, and resistance to tetracycline, cefotaxime, amoxicillin+clavulanate, and amikacin. β-hemolytic *streptococci* had a high sensitivity to cefotaxime, amoxicillin+clavulanate and azithromycin, and high resistance against tetracycline, amikacin, and gentamicin. Both species, plus *Staphylococcus* spp., showed resistance to tetracycline.

**Table 2:** Effects of antibiotics in semen extenders on sperm quality.

Reference	Antibiotics	Effect on Sperm Quality
[65]	Amikacin, gentamicin, potassium and sodium penicillin, polymixin B, streptomycin, and ticarcillin	Total motility, progressive motility and rapid motility were lower when polymixin B was added than for the other antibiotics
[66]	Gentamicin	Adverse effect on sperm motility and velocity. These authors concluded that the presence of gentamicin could affect sperm function during cooled storage
[67]	Llincomycin and spectinomycin	Sperm DNA fragmentation index was greater in the samples containing these antibiotics than in those without. Sperm motility, membrane integrity and mitochondrial membrane potential were not different between semen samples with and without lincomycin and spectinomycin
[64]	Potassium penicillin G-amikacin disulfate, ticarcillin disodium-potassium clavulanate, piperacillin sodium/tazobactam sodium, or meropenem	Slight differences were detected for sperm motility and kinematics and in chromatin integrity when various antibiotic combinations were used
[68]	Adding amikacin sulfate and potassium penicillin G to INRA-96^®^ extender (already contains penicillin, gentamicin and amphoteracin-B)	Reported to increase the antimicrobial effect without having an adverse effect on sperm motility

## Data Availability

Data sharing not applicable. No new data were created or analyzed in this study. Data sharing is not applicable to this article.

## Supplementary Materials

The following are available online at https://www.mdpi.com/article/10.3390/ani11113035/s1, Supplementary file: Literature searches.
