# Supplementary material for: Antimicrobial Resistance in Equine Reproduction
Source: Animals (Basel). 2021 Oct 22;11(11):3035. doi: 10.3390/ani11113035 (PMC8614435; doi:10.3390/ani11113035)
Supplement: Supplementary file 1 [file animals-11-03035-s001.zip › animals-1285458-supplementary.pdf]

Supplementary file 1: Literature searches

Several literature searches were carried out in the production of this article, using databases such as Google Scholar, PubMed and Web of Science. In most instances, Google Scholar was the primary source and revealed the most articles. PubMed resulted in the same selection but was more limited.

The keywords were

(1) antibiotic or antimicrobial resistance (e.g., antibiot* or antimicrob* resistan*)

(2) equine or horse (equin* or horse*)

(3) vagina or uterus (vagin* or uter*)

In addition, searches were made of these databases for specific items, such as:

Minimal contamination techniques for breeding mares

- Pharmacokinetics, uterus, mare (add in specific antibiotics)
- Equine, endometritis, treatment
- Bacteria, sperm quality
- Antibiotics in semen extenders and sperm quality
- Alternatives to antibiotics, semen extenders

In all cases, exclusion criteria were publications in languages other than English, publications before 1970, and publications deemed to be of poor quality.
